# 

**Published:** 1820-09-01

**Authors:** 


### LECTURES, &c.

*Medical Botany*. Botany being now so essential a part of the Medical Gentleman's Education, we are gratified to find men of talent and experience engaged in conveying the necessary knowledge through the medium of Lectures, as well as printed works. Among the Lectures on Botany, we beg leave to draw the attention of the medical student to those of Dr. Emerson, who facilitates the study by arranging the matter under the following five heads; viz.

1. Of the parts of fructification, on which generic character is founded.
2. Illustration of the Linnæan system.
3. Of the anatomy and physiology of plants.
4. Of the elements, and immediate principles of vegetables.
5. Of the natural orders, and of the medicinal plants included in them.

In this part of the Course, the *analogy* between the *forms* and *properties* of plants is explained, agreeably to the latest discoveries of scientific Botanists. These Lectures also form a principal feature in the Autumnal and Winter Courses of Materia Medica. It is Dr. Emerson's intention to publish a text-book in the course of a short time.*

---

Mr. Davies, Member of the Royal College of Surgeons, Surgeon in the Army, Surgeon to the Household of his Royal Highness the Duke of Cumberland, and Surgeon-Man-Midwife to several Lying-In Charities, &c. &c. will commence his Autumn Course of Lectures on the Theory and Practice of Midwifery, and on the Diseases of Women and Children, in the Waiting Room of the Royal Westminster Infirmary for Diseases of the Eyes, Marylebone-street, Piccadilly, on Tuesday October 3d, at a quarter-past Ten o'Clock in the Morning.—Medical Officers of the Navy, the Army, and the Ordnance, will be admitted to attend these Lectures, on presenting a recommendation from the heads of their respective departments to Mr. Davies, at his house, on Monday, Wednesday, or Friday Mornings, before Eleven o'Clock.

---

*Royal Dispensary for Diseases of the Ear, Dean Street.* Mr. Curtis will commence his next Course of Lectures on the Anatomy, Physiology, and Pathology of the Ear, and on the Medical Treatment of the Deaf and Dumb, on Monday, October the 2d. For particulars apply to Mr. C. at his house, No. 2, Soho Square.

---

*St. George's Medical, Surgical, and Chemical School.* The Courses of Lectures will commence the First Week of October, *Namely*, 1. On the *Laws of the Animal Œconomy and Practice of*

---

* We beg leave to correct an error in our Number for April last, p. 644, where we stated Dr. Emery to be Physician to the Dispensary for Diseases of the Skin. We meant Dr. Emerson, who is one of the principal medical officers of that very useful establishment, which we again recommend to the patronage of the profession.

*Physic;* by GEORGE PEARSON, M. D. F. R. S. Senior Physician to St. George's Hospital, &c. &c.—2. On Therapeutics, with Materia Medica; by GEORGE PEARSON, M. D. &c.—3. On Chemistry, at the Royal Institution Laboratory; by W. T. BRANDE, Professor R. I. and Sec. R. S. &c. &c.—4. On the Principles and Practice of Surgery; by B. C. BRODIE, F. R. S.; Assistant-Surgeon to St. George's Hospital, &c. &c.

*Note.* Sir EVERARD HOME will deliver Lectures on Surgery to the Pupils of St. George's Hospital.

---

*Middlesex Hospital.* The Medical Lectures will commence on Monday the 9th of October. Theory and Practice of Physic, by Dr. Southey, on Mondays, Wednesdays, and Fridays, at Nine in the Morning; Chemistry, by Dr. Ashburner, on Tuesdays, Thursdays, and Saturdays, at Nine in the Morning; Materia Medica, by Dr. Southey and Dr. Ashburner, on Tuesdays and Saturdays, at Eight in the Morning; Midwifery, and the Diseases of Women and Children, by Dr. Merriman and Dr. Ley, every Morning, at half-past Ten.—Further Particulars may be learnt, on Application to Mr. Biddel, Apothecary, at the Hospital.

---

*Theatre of Anatomy, Great Windmill Street.* The Lectures on Anatomy, Physiology, Pathology and Surgery, by Mr. WILSON, and Mr. CHARLES BELL, Surgeon to the Middlesex Hospital, will commence on Monday Oct. 2, at two o'clock. The Demonstrations in the rooms will be given by Mr. SHAW, who will assist the Students during the morning.

☞ *Mr. Shaw will this Season take a House Pupil.*

---

Dr. JOHNSON's little Work "on Civic Life, Sedentary Habits, and Intellectual Refinement," has been re-published in Philadelphia.

---

On the 1st of October will be published, Outlines of Midwifery, developing its Principles and Practice; with illustrative Lithographic Engravings, in 1 Vol. 12mo. Principally designed for Students. By J. T. CONQUEST, M. D. F. L. S. &c.

---

### IN THE PRESS.

A Dissertation on the Treatment of Local Affections of Nerves; to which the Jacksonian Prize of the College of Surgeons was adjudged. By JOSEPH SWAN, Esq. Surgeon to the Lincoln County Hospital.

Essays on the Employment of Caustic. By W. J. HIGGINBOTTOM, Surgeon, in Nottingham.

### *Books received for Review since last Quarter.*

A Dissertation on Infanticide, in its Relations to Physiology and Jurisprudence. By WILLIAM HUTCHINSON, M. D. F. L. S. Octavo, pp. 99. London, 1820.

---

A short Account of some of the principal Hospitals of France, Italy, Switzerland, and the Netherlands; with Remarks upon the Climate and Diseases of those Countries. By H. W. CARTER, M. D. F. R. S. Ed.; one of Dr. Radcliffe's Travelling Fellows from the University of Oxford. Octavo, pp. 255. London, 1819.

---

Memoire sur l'Utilité des Pièces d'Anatomie Artificielle Chirurgicale. Par J. F. AMELINE, &c. &c. &c. Professeur d'Anatomie a Caen, &c. 1 Vol. Octavo, pp. 89. Paris, 1819.

---

Reflexions Critiques sur un Ecrit de M. Chomel, ayant pour Titre: "de l'Existence des Fièvres." Par TH. DUCAMP, M. D. &c. &c. 1 Vol. Octavo, pp. 82. Paris, 1820.

☞ *An able defence of the doctrine of Broussais, with some judicious modifications of that distinguished physician's theory of fever.*

---

Practical Essays on Strictures of the Urethra, and Diseases of the Testicles, including Observations on Fistula in Perinæo and Hydrocele. Illustrated by numerous Cases, and an Engraving; and prefaced with some Remarks on Life and Organization. By ROBERT BINGHAM, Fellow of the Royal College of Surgeons. 1 Volume, Octavo, pp. 357, with a Plate. London, 1820.

---

A Treatise on Inflammation of the Mucous Membrane of the Lungs; to which is prefixed, an Experimental Inquiry respecting the Contractile power of the Blood-Vessels, and the Natnre of Inflammation. By CHARLES HASTINGS, M. D. Physician to the Worcester Infirmary; late President of the Royal Medical Society, Edinburgh, &c. 1 Vol. Octavo, pp. 420. London, 1820.

---

Advice and Maxims for Young Students and Practitioners of Medicine; with Remarks on the Pulse. By DANIEL JOHNSON. Octavo, sewed, pp. 32. London, 1820.

☞ *This little pamphlet contains much good advice, and many ingenious observations.*

---

General Principles of Psycho-Physiology. By EDWARD SMITH PEARSON. Octavo, pp. 62. London, 1820.

☞ *An ingenious and laudable attempt to prove the immateriality of mind, even on physiological principles.*

---

Proposal for establishing, in Edinburgh and other Towns, a Newly Improved Apparatus for the Application of the Vapour of Water, Sulphur, and other Medicinal Substances, found so efficacious in the Cure of Rheumatism, and Diseases of the Skin; with a Paper upon the Subject, which has been submitted to the perusal, and received the approbation of Dr. Hamilton, Sen. Dr. Gregory,

Dr. Barclay, Dr. Farquharson, and Mr. Bryce. By WILLIAM SCOTT, Esq. Octavo, sewed, pp. 56. Edinburgh, 1820.

☞ *This is the first number of a work which is designed to be published, not exactly periodically; but as materials may accumulate or present themselves. The establishment for vapour baths, in Edinburgh, has been got up under the direction of Mr. Scott, and, we are informed, is patronized by the faculty, and likely to be generally encouraged by the public at large. In the pamphlet, herewith announced, Mr. Scott has traced the origin and importance of baths from Hippocrates downwards; and gives numerous extracts from, or rather an analysis of Dr. De Carro's work* "Sur les Fumigations Sulfureuses," *lately published in Vienna. The pamphlet contains many judicious and original observations besides, and is deserving of the reader's attention. The Institution itself is, we conceive, entitled to the grateful patronage of all classes in the Scottish metropolis.*

⁂ A few Medical Students will be received as Boarders at the Establishment in Drummond Street.

---

Revue Medicale, Historique et Philosophique. No. III. for May, 1820.

☞ *This work, consisting of about* 170 *pages each number, is published every two months, containing extensive reviews of medical works, French and foreign. It is somewhat on the plan of our own Journal; but more addicted to criticism than analysis, a characteristic feature of the French journals in general. It is not, therefore, so useful to the English reader as it otherwise would be, did it adopt the analytical course; yet still it is by far the best* Review *published in Paris, and as such we can recommend it to our brethren. Extracts from the work will regularly appear in our Journal.*

---

Cases of Hydrophobia. By GEORGE PINCKARD, M. D. Octavo, sewed, pp. 38. London, 1819.

---

A Synopsis of the various kinds of Difficult Parturition, with Practical Remarks on the Management of Labours. By SAMUEL MERRIMAN, M. D. F. L. S. Lecturer on Midwifery; Physician-Accoucheur to the Middlesex Hospital, and to the Parochial Infirmary of St. George's, Hanover Square; and Consulting Physician Accoucheur to the Westminster General Dispensary. *Third Edition,* with considerable Additions; and an Appendix of Illustrative Cases and Tables. 1 Vol. Octavo, pp. 324, and five Plates. London, 1820.

---

A Treatise on Verminous Diseases, preceded by the Natural History of Intestinal Worms, and their Origin in the Human Body. Translated from the Italian of VALERIAN LEWIS BRERA, Professor of Clinical Medicine in the University of Pavia. By JOHN G. COFFIN, M. D. of Boston in America. 1 Vol. Octavo, pp. 363, with Plates. Boston, 1817.

☞ *We return Dr. Coffin many thanks for this very valuable volume, which we shall take an early opportunity of fully analysing, for the*

*benefit of the British medical public; as there is no work on the subject of Intestinal Worms and Verminous Diseases, in our language, at all to be compared with it for science and utility.*

---

Discourses on Cold and Warm Bathing; with Remarks on the Effects of drinking Cold Water in Warm Weather. By John G. Coffin, M.D. of Boston, United States. 8vo. pp. 75. Boston, 1818.

☞ *These discourses contain many excellent observations on bathing, that cannot be too generally known by all classes of society.*

---

A Toxicological Chart; in which is exhibited, at one View, the Symptoms, Treatment, and Modes of detecting the various Poisons, Mineral, Vegetable, and Animal, according to the latest Experiments and Observations. [Most respectfully dedicated to the Royal Humane Society.] By a Member of the Royal College of Surgeons, London. Two Sheets, Price 1s. : 6d.; or on Pasteboard, 2s. : 6d. London, 1820.

☞ *The readers of our Quarterly Series will remember that we suggested a Chart of this kind in our fourth number. That suggestion led to the present attempt; and, although some slight objections might be made to certain passages in the column of tests, (a fruitful source of disputation by the bye) yet we consider the Chart as very fairly executed, according to our present state of knowledge on the subject, as published in professed toxicological writings. We have placed the Chart in our own library, and we think no medical practitioner should be without it. It should also be hung up in the shops of all chemists and druggists, as well as in the surgeries of general practitioners. It is not to save chemical or toxicological study, but to prevent a moment's delay, when every moment is precious, and the life of a fellow creature at stake.*

---

An Essay on Mercury; wherein are presented Formulæ for some Preparations of this Metal, including Practical Remarks on the safest and most effectual Method of Administering them, for the Cure of Liver Complaints, Dropsies, Syphilis, and other formidable Diseases incident to the Human Frame; being the result of long experience and diligent observation. By David Davies, M. D. Member of the Royal College of Physicians and Surgeons, London; and Senior Physician to St. Peter's Hospital, Bristol. Octavo, sewed, pp. 35. 1820.

---

Observations on Variolous Inoculation and Vaccination; in a Letter to a Friend. With an Appendix, containing some Remarks on the Extension of the Small-pox, in the Town of Melksham and its Vicinity. By J. F. Hulbert, Member of the Royal College of Surgeons, London, &c. &c. Octavo, sewed, pp. 53. London, 1820.

☞ *A well written eulogium on vaccination, and a dignified remonstrance against the practice of variolation.*

Popular Observations on Regimen and Diet; in which the Nature and Quantities of our common Food are pointed out and explained; together with Practical Rules and Regulations in regard to Health, adapted to various Situations and Circumstances, from Infancy to Old Age. By John Tweed, Surgeon, &c. Bocking, Essex. 1 Vol. Octavo, pp. 248. London, 1820.

☞ *The title page fully explains the nature and design of the work, which enters more minutely into the detail of regimen and diet, and in a more profitable manner for the popular reader, than any other work of this class. To use the author's own words—" the valetudinarian will find in it rules which promote his recovery; and he who enjoys good health may learn to preserve it."*

---

Dissertatio Medica Inauguralis Quædam de Physiologia et Effectibus Electricitatis Orientia, complectens. Per Franciscum Moran, M. D. Hibernum. 8vo. p. 25, Ed. 1820.

☞ *A well written, and practical little Thesis, which is creditable to its Author.*

---

*Medical School, St. Bartholomew's Hospital.* The following Courses of Lectures will be commenced at this Hospital, on Monday October the 2d, at two o'clock. On the Theory and Practice of Medicine, by Dr. Hue; on Anatomy and Physiology, by Mr. Abernethy; on the Theory and Practice of Surgery, by Mr. Abernethy; on Chemistry and Materia Medica, by Dr. Hue; on Midwifery, by Dr. Gooch; Practical Anatomy, with Demonstrations, by Mr. Stanley.—Further particulars may be obtained, by application to Mr. Wheeler, Apothecary to the Hospital; or, of Mr. Anderson, Medical Bookseller, 40, West Smithfield.

---

Mr. Edward Grainger, jun. will commence his Autumn Course of Lectures on Anatomy and Physiology, on Tuesday the 3d of October, 1820, at twelve o'clock, at his Rooms, 13, St. Saviour's Church Yard, Southwark. The Lectures will afterwards be delivered daily, at eleven o'clock. Terms: Lectures and Dissections, single Course, 3l. 3s.; Perpetual, 10l. 10s.—Three Courses are given in the Year, beginning in October, January, and June. A few gentlemen are received as House Pupils.

Preparing for Publication, A concise System of Anatomy, for the Use of Medical Students; by Edward Grainger, junior, Member of the Royal College of Surgeons, and Lecturer on Anatomy and Physiology.

---

Lectures on the Principles and Practice of Midwifery, on the Diseases of Women and Children, and on some Points of Medical Jurisprudence. By John T. Conquest, M. D. F. L. S. Member of the Royal College of Physicians of London, and Physician-Accoucheur to the City of London Lying-In Institution.—Pupils, when properly qualified, will be supplied with several Cases of Labour gratuitously.—Terms: One Course, 3l. 3s.; Two Courses, paid for at once, 5l. 5s.; Perpetual, 8l. 8s.; Private Course to One Pupil, 10l. 10s.—Gentlemen who are Assistants, and Apprentices, or the Sons of Medical Men, become Perpetual on paying 5l. 5s.—Four Courses are given in the Year, commencing on the First Monday in October and January, at a quarter-past five o'clock in the evening; and on the first Monday in April and July, at a quarter-past eight o'clock in the morning. Further particulars may be known of Dr. Conquest, at his house, No. 4, Aldermanbury Postern, where the Lectures are delivered.

---

*To Lecturers, Authors, &c. &c.*

As all Miscellaneous Notices in this Journal are printed beyond the Limits of the Review, and consequently at the Editor's own Expence; and, moreover, as Government charges every Announcement of Lectures as an Advertisement, it is hereby signified, that no Announcement of Lectures will, in future, be received, unless accompanied by *Seven Shillings;* and if they exceed Ten Lines, One Shilling on each Line beyond that Space.

---

The Medico-Chirugical Review, and Journal of Medical Science, offers the following facilities for Advertising; viz. 7 Lines and under, 7s.—Fourth of a Page, 10s. 6d.—Half a Page, 18s—Three Fourths of a Page, 25s—One Page, 30s.—Bills, Catalogues, &c. stitched in (1250) 24s 6d.—Bills or Catalogues (1250 in No.) to be sent to Mr. Sanders, Little Shire Lane, Temple Bar.—All Advertisements and Bills must be sent eight days before publication days, which are as follow, viz. on the 1st of March, 1st of June, 1st of September, and 1st of December.

---

Some Subscribers' Names arrived too late, the List having been put to press. They will appear in our next.

---

Our Readers will perceive that we have exceeded the regular boundaries of the Journal, this Quarter, by twenty pages, besides printing a portion of the work in small type. The press of important articles prevented us inserting a very long "Supplimental Review," which will appear in our next without fail.

---

All the periodical *Transactions*, published in this Country, will be regularly and minutely analysed in this Review; two gentlemen, of distinguished talents and rigid impartiality, having undertaken that particular branch of the work, so as to effect the object in the fullest manner, and without any precipitation or negligence.

Dr. John Latham, Pres. of the R. C. of Physicians, Harley-st.
Sir H. Halford, Bt. Curzon-street, May Fair.
Professor V. L. Brera, of the University of Pavia.

Barton, Francis, M. D. and Mem. Extraord. of the Royal Physical Society of Edin. Whitby
Berry, Mr. Surgeon, Windsor, 2 Copies
Brookes, B. Esq. Surg. Reading
Brookes, Mr. Surgeon, Wenlock
Burgoyne, Mr. Surgeon, Hon. East India's Co's Service
Caddy, Mr. Sur. Great Torrington
Cockburn, Dr. Mark, Dunse
Christie, Dr. Thos. Cheltenham
Corsham Medical Book Society, 2 Copies
Curtis, John, M. D. Cowley
Carver, Walter, Esq. Surg. 18th Regt. (spelled by mistake Surgeon Larver.)
Dean, Mr. Surgeon, Standress
Dempster, Mr. Assistant Staff Surg. Land Guard, Harwich
Emery, Dr. Physician to the Forces
Foote, Mr. Surg. Tavistock-st.
Forster, Mr. Stafford-place
Fowke, Mr. Surgeon, Wolverhampton
Gallup, Dr. Joseph A. Woodstock, United States
Gaitskell, Mr. Surg. Rotherhithe
Gibney, Dr. Brighton
Gordon, Mr. Oxford-court
Grimstone, E. Esq. Uxbridge
Griffith, Mr. R. Flag-Surgeon, Mediterranean
Graham, Mr. Oxford-court
Greenhead, Mr. late of Jamaica
Harrison, Dr. R. Fel. of the Roy. Coll. of Phys. Sackville-street
Harrold, J. C. Esq.
Hill, Mr. D. Great Russel-street
Huntley, Mr. L. Surg. Brixton
Hulbert, Mr. J. F. Mem. Roy. Coll. Surg. London, Melksham
Hunter, Dr. J. Lees, Wetherby
Junior, or Medico-Chirurgical Society, Portsmouth
Lickorish, Mr.
Lloyd, Mr. James, Licentiate of the Roy. Coll. of Surg. Ireland
Madden, Mr. J. Mallachi, R. N.
M'Calman, Mr. Duncan, Assistant Surgeon, Bengal
Miln, Dr. Res. Physician, Naples
Mussey, Dr. R. D. Professor of Physic, Dartmore College, New Hampshire
Norwood, J. Esq. Ashford, Kent
Nutt, James, Esq. R. N.
Page, John, Esq. Dartmouth
Perkins, John, Esq. Rue d'Orangerie, Brussels
Porter, Mr. Surg. Bishopsgate-st.
Probart, Mr. Surg. Hawarden, Flintshire
Pomeroy, Dr. Burlington Vermont, United States
Royle, Mr. Surgeon, Chester
Short, Dr. P. M. O. St. Helena
Settle, Joseph, Esq. Surgeon, Plymstock
Sexty, Mr. R. Sur. Hammersmith
Shattuck, Dr. George C. Vermont County, United States
Seymour, Mr. John Gouldin, Bishop's Waltham, Hants
Steel, Mr. Tower Hill
Somerville, Dr. Hanover-square
Stewart, Dr. 71st Regt. Brussels
Stuart, Dr. Bute, Wisbeach, Cambridgeshire
Stilwell, Mr. Walton on Thames
Thomas, Mr. Surgeon, Hatfield
Watling, T. F. Esq.
Willson, Sir Alexander, M. D. Mead House, near Chepstow
Yonge, Dr. Henrietta-street, Cavendish-square

---

*Changes of Residence, Designation, Appointments, &c. &c. &c.*

Dr. Uwins, from Thaves Inn to Bedford-row.
Mr. John Shaw, from Soho-square to 1, Albany, Piccadilly.
Dr. Vetch, from Edgeware-road to Regent-street.
Dr. A. P. W. Philip, from Worcester to Hanover-square, London.
Dr. James Johnson, from the Albany to Spring Gardens.

